# The social values of newly arrived immigrants in Sweden

**DOI:** 10.1371/journal.pone.0278125

**Published:** 2022-11-22

**Authors:** Andrey Tibajev, Irina Vartanova, Soorej Puthoopparambil, Birgitta Essén, Pontus Strimling

**Affiliations:** 1 Institute for Futures Studies, Stockholm, Sweden; 2 Department of Women’s and Children’s Health, Uppsala University, Uppsala, Sweden; University of Macerata, ITALY

## Abstract

Concerns have been raised that immigrants coming to Europe bring fundamentally different social values, affecting the more liberal receiving societies negatively. However, the topic of immigrants’ social values is understudied, and much research studies only one issue at a time, lacking a systematic approach to compare immigrants and native-born across issues. We study the social values of immigrants in Sweden using a large sample of newly arrived immigrants and their opinions on 35 different moral issues. Our results indicate a large heterogeneity across different issues, with, on average, a general tendency towards liberal social values among immigrants. We find that individual characteristics are more important than characteristics of the country of origin in explaining variation of social values between immigrants. Religiosity has the largest effect, with more religious individuals having more conservative stances. Using external data sources, we compare immigrants with native-born regarding both average positions on different issues, and the correlation between issue positions. Compared with the native-born, immigrants have, on average, somewhat more conservative values, but the underlying values structure is the same.

## Introduction

### Perceptions of the social values of immigrants

During the last decades, Europe has experienced a large immigration from countries in the Middle East, North Africa, and Central Asia [1]. With this immigration, concerns have been raised—especially targeting refugees and Muslim immigrants [2–5]—about how it will change receiving societies. One particularly salient feature of such concerns is the fear that these immigrants have too disparate, or fundamentalist, social values [6–8].

Subsequent to these concerns, some politicians in Europe have proclaimed the end of multiculturalism [9] and the previous expansion of multi-cultural policies, i.e., policies that recognise, encourage, and aid cultural diversity, have stagnated [10, 11]. Instead, many Western countries have increased civic integration measures, imposing requirements or tests for residency and citizenship [12], resting on a political critique against multi-culturalism and a fear of disloyalty among the immigrant population [13, 14]. Whether adopted as a retrenchment of an already restrictive citizenship regime or as a counterbalance to inclusive citizenship [15], this so-called civic turn in immigrant integration is built on the idea that a society needs shared values among its members to function properly [16, 17]. Immigrants are thus automatically defined as different, in need of being educated, and obliged to prove that they belong before they can be accepted as full members of society.

The perceived threat of the social values of immigrants have spurred the rise of radical right-wing anti-immigrant parties, that no longer build their political programmes on non-European immigrants being genetically different, but rather because they differ culturally [18]. According to these parties, immigrants have a fundamentally different value system compared to the majority native-born European population, and since culture is considered to be an essential and invariant quality of individuals or groups according to this perspective, high volumes of immigration are perceived to threaten the European, or Western, values system that the receiving societies are built on [19, 20].

About 20 percent of the Swedish population are foreign born, i.e., immigrants [21]. Much like the rest of Europe, Sweden has seen a rise of an anti-immigrant radical right-wing party as well as adoption of anti-immigrant rhetoric among the mainstream parties [22, 23]—a rhetoric that is concentrated on culture, values, and belonging [24, 25]. Sweden is also moving towards introducing civic integration requirements in the form of a language and knowledge test for permanent residency [26].

However, despite these concerns and political measures, there are clear gaps in the knowledge of the social values of immigrants and whether, in fact, they are fundamentally different from those of the native-born. Although the topic has been studied at some length, individual studies tend to include only a few issues [e.g., a short gender equality index 27, 28] or even just one issue [e.g., homosexuality 29–34]. We believe that claims about the social values of immigrants, and how they compare to those of the native-born, require more robust evidence, calling for a broader and more systematic approach.

### Measuring social values

We define social values as the orientation of an individual’s normative and moral perspective on society and the behaviour of others. This is similar to the use of the term cultural values in Norris and Inglehart [35], and with an emphasis on the social aspect of value formation and direction [36]. The concept is operationalised and measured by survey questions asking about opinions, or issue positions, on 35 specific moral issues.

When comparing the social values between two different populations, such as immigrants and native-born, we additionally distinguish between the difference in average positions on issues, and different opinion structures. For example, assume a native-born population with 60% support for abortion and 80% support for divorce, and three hypothetical immigrant populations with an average support for abortion and divorce of 20% and 40%, 30% and 30%, or 40% and 20%, respectively. In all three cases, the average difference is 40% between the immigrant and native-born populations for both issues. However, the three immigrant populations are clearly not equal in relation to the native population.

We use the concept of opinion structures as a relative term to understand and compare the values of different populations. We measure opinion structures by correlating the average issue positions of two populations with each other. In the first immigrant population above, the correlation between immigrants and native-born is one; they would have the same opinion structure (despite immigrants on average being more against both abortion and divorce). In the second population, there is a zero correlation between the average positions of immigrants and native-born, and we would conclude that the populations have unrelated opinion structures. In the third example, the correlation minus one, i.e., completely opposing opinion structures.

The distinction between average positions and opinion structures is important when comparing immigrants and native-born (a similar argument regarding the need for a correlation analysis when studying different populations, and the use of a more complex measure, in [37]). From previous research, we know that many immigrants and immigrant populations that come to European and Western countries differ on average positions on different issues, compared with the native-born population in [35]. However, a difference in average positions is not sufficient to conclude that immigrants have fundamentally different social values. To reach such a conclusion, we argue, immigrants must either have oppositional positions on several issues, or have a different opinion structure, compared with the native-born. This is what we intend to study for a broad set of issues.

### Aim and research questions

The aim of this paper is to describe the social values of newly arrived immigrants in Sweden, and to compare them with the social values of native-born Swedes. Sweden is typically one of the most extreme countries regarding social values in country-comparisons, e.g., being in a corner on the so-called cultural map [38]. It makes the Swedish setting particularly interesting for the study of the social values of the immigrant population, and how these values compare to those of the native-born.

Using a large-scale survey of newly arrived immigrants in Sweden, and comparing with external data sources for the native-born, we will answer three research questions:

1. What are the social values of newly arrived immigrants?
2. How do their social values correlate with the characteristics of the country of origin and with the individual characteristics?
3. How do their social values compare to the social values of native-born Swedes?

In addition to presenting the social values of a large sample of newly immigrants in Sweden, we make two important contributions to existing literature on the social values of immigrants. First, we study a broad array of issues covering values related to, inter alia, sexual and reproductive rights, gender equality, xenophobia, religion, violence. This adds to previous research that has mostly concentrated on a few or even just one issue at a time. Second, our inclusion of opinion structures provides a novel way of analysing the correlation of social values between two populations, and adds a new understanding of whether immigrants and native-born have fundamentally similar or different social values.

### Explaining variation in immigrants’ social values

The study’s aim is explorative rather than hypotheses-driven. When analysing the variation in immigrants’ social values (RQ2), we include characteristics that are prominent in public debates regarding the values of immigrants as well as important results in previous research.

Previous research has confirmed that immigrants have, on average, differing values compared with the native-born population, often falling somewhere in between their old and new home countries [30, 33–35]. While immigrants are not entirely representative of their countries of origin, the countries in which they grew up will naturally have an impact on their values [39]. To study newly arrived immigrants, who have had limited exposure to the values in their new country, thus offers the possibility to measure immigrants’ values before acculturation in their new country and possible subsequent changes in values.

To measure the influence of origin, we include three characteristics on the country-of-origin level that previous research has established to be important when explaining variation in values across the world: modernisation theory, moral foundations theory, and the specific path of Muslim countries. First, according to modernisation theory, the level of socioeconomic development in a country will affect its citizens’ social values by diverging them from seeking to remedy economic scarcity or survival, to self-expression, spurring more tolerant post-materialistic values [38, 40, 41]. Second, based on moral foundations theory, opinion change towards more liberal values happens with the exchange of arguments and opinions between individuals [42]. In a society without a free expression of speech, public opinion will be hampered in this change, resulting in, on average, more conservative social values. Third, we include percentage of Muslims in the origin population, following the research that emphasises different cultural traditions and the divide in social values between Western and Muslim countries, with people in the latter being more conservative and the importance of origin-country traditions on immigrants’ social values [35, 43, 44]. Including percentage of Muslims is additionally in accordance with literature emphasising different barriers to diffusion of information and norms across populations [45].

On the individual level, we include gender and level of education as basic demographic characteristics that have been shown to correlate with social values in general, and where women and more highly-educated individuals exhibit more liberal social values [46–48].

We also include individual grounds for residence, specifically, refugees vis-à-vis other categories. Refugee status can be connected to social values in at least two ways. First, refugees may have to flee their countries of origin for reasons linked to social values, e.g., belonging to a religious minority or being in opposition to the government. Second, immigrants that are discriminated or exposed to hostility in their destination countries—as noted, refugees fall into this kind of category of immigrants—can react buy holding on to their original, more conservative, social values or even develop an opposition culture to the mainstream [32, 49–51]. However, this second mechanism relies on (relative) changes over time and since we only study immigrants recently arrived in Sweden it might be of lesser importance.

Finally, we include individuals’ religion, separating the measures of the intensity of belief (religiosity), and self-described religious affiliation (religion). Both can have an effect on social values in that religions may prescribe specific norms and moral rules regarding behaviour while religiosity captures the degree to which an individual practises their religion and thus adheres to these norms [52]. In previous research, both religion and religiosity, have shown to be strong predictors of immigrants’ social values, with Muslim and more religious individuals being more against gender equality [27, 28], homosexuality [30–32, 46, 53], premarital co-habitation [52], and hold more conservative views on sexual and reproductive rights [35, 54].

## Materials and methods

### The Swedish immigrant values survey

Data for the social values of immigrants comes from the Swedish Immigrant Values Survey (SIVS). The research project, survey, and data collection have undergone ethics vetting and been approved by the Swedish Ethical Review Agency (decision numbers 2019–04146 and 2020–05269). At the start of the digital survey, all respondents were informed of the purpose of the research project and the survey, their participation and rights, how data is handled according to the European General Data Protection Regulation, and whom to contact for additional information. Before they could start the survey, all respondents had to give their written informed consent including consent to participate in the research project, that their information will be saved, and that they will be contacted in the future for a follow-up study.

Survey responses were gathered among students of Swedish for Immigrants (SFI) during 2020–2021. SFI is Swedish language education for adults provided by the municipalities. It is an institutionalised part of the public integration programme for refugees and family migrants, and additionally available for almost all other immigrants with a residence permit, free of charge. When designing a study of a hard-to-survey population, such as newly arrived immigrants, the trade-off between cost, on the one hand, and coverage and representativeness on the other, becomes central [55]. Being an institutionalised point of entry into the Swedish society, SFI is a well-suited site to recruit respondents, covering a large proportion of newly arrived immigrants at a reasonable cost (for a similar approach to recruit newly arrived immigrants, see [56]).

The survey was digital (online) and available in Arabic, Dari, English, Somali, Swedish, and Tigrinya. Data was collected in two modes. First, five municipalities collected data among their students—initially in classrooms using tablets but later switching to invitations by e-mail and internal communication systems due to the shift to online education because of the covid-19 pandemic. Second, we collected e-mail addresses of all students who attended SFI in 2020 or 2021 from 101 municipalities, following a confidentiality assessment from each municipality and in accordance with approved ethics review by the Swedish Ethical Review Authority, and the Public Access to Information and Secrecy Act (SFS 2009:400). An e-mail invitation with a link to the survey was sent to all gathered e-mail addresses, in total 63,380 invitations. The two modes covered 37% of all municipalities in Sweden, containing approximately 71% of all SFI students. More on the survey and data collection can be found in the documentation and codebook for the survey [57].

Though the data gathering modes enabled a large sample size of the target population of newly arrived immigrants, they confer a limitation regarding statistical inference. We have weighted all results—calibrating for gender, education, and region of origin including interactions between all variables—to make the sample more representative of newly arrived immigrants in Sweden. This procedure rests on the assumption that the different subgroups in the sample will have similar results on the outcome variables as their equivalents in the population [58].

### Analytical sample

Since the aim of the study is newly arrived immigrants, we only include respondents who have been in Sweden for a maximum of five years in the analytical sample, a total of 3360 respondents. This to avoid individuals who attend SFI after a long time in the country, as these individuals could be self-selected on important unobservable variables that correlate with social values. The item non-response rate, i.e., respondents skipping individual questions, is, on average, only four percent. However, the SIVS is a relatively long questionnaire; The median time for completion was 33 minutes. Respondents could drop out at any point and, as expected, there was a gradual increase in drop-off. To avoid comparing different samples across issues, we opted for case-wise deletion on all included variables in the forthcoming analyses. This excluded an additional 1186 respondents, leaving an analytical sample of 2174 individuals who had lived in Sweden for, on average, two years at the time of the survey.

### Inclusion and coding of issues

We have included 35 issues connected to social values from the SIVS, described in S1 Table. For native-born Swedes, seventeen of the included issues are recorded from various external data sources: the ADL GLOBAL 100 [59]; the European Social Survey [60]; the European Values Study [61]; the International Social Survey Programme (ISSP) [62, 63]; the Society, Opinion and Media Survey (SOM) [64]; the World Values Survey (WVS) [65].

We present the positions on the included issues on standardised scales ranging from zero to one, regardless of the number of answer categories in the corresponding survey question (original categories and full distribution can be found in [57]). The mean for each issue can thusly be interpreted as the average position for a specific population.

A conventional way of presenting data on values and opinions is to recode included issues so that the there is a common direction, i.e., as an operationalisation of some underlying concept. To decide the directions, some studies use an approach with statistical correlation analysis and post-hoc labelling the different directions to e.g., secular-rational values [38] or liberal social attitudes [47]. In this study, we define liberal, as opposed to conservative, positions for an issue based on the moral arguments that underpin them. According to Moral Foundations Theory, moral opinions are underpinned but a finite set of foundations such as fairness or loyalty [66–68]. One key aspect of the theory is that individuals who define themselves as liberals only find some arguments relevant, while conservatives accept arguments connected to all foundations. Building on this notion, a liberal position on an issue can be defined as the position that is more strongly connected to the arguments that are universally relevant: harm reduction, freedom, and fairness [42, 69].

In the SIVS, respondents answered questions about what moral arguments that would be relevant for people either for or against an issue, e.g., for or against finding abortion justifiable, regardless of their own opinion. Following previous research, the position with the most respondents who answered that it is based on arguments about harm reduction, fairness, and freedom—a so-called liberal argument advantage—is coded as the liberal position. Results are available in S1 Fig. Comparing with Eriksson, Vartanova [70], who also use moral arguments, our results overlap to a large degree in the definitions of liberal/conservative direction. There is also a great overlap with the conceptualisations based on correlation analysis [38, 47].

Unfortunately, eight issues have not connected moral foundations questions in the SIVS. We have coded three of the issues—female genital pricking (against), if men make better political leaders than women (against), and if parents can decide who their children should marry (against)—based on three related issues, if it is acceptable to investigate girls’ virginity, that a university education is more important for a boy than a girl, and if parents can decide if their children should wait with sex before marriage, respectively. The remaining five issues are connected to xenophobic attitudes, which we have coded so that the non-xenophobic position is equal to the liberal position, as xenophobia has been shown to be connected to right-wing authoritarianism [71].

Note that these directions mainly are a way to present the data. For the analysis of the average values of immigrants and native-born, direction doesn’t inherently matter. However, for the analyses on opinion structures of different populations, the choice of what is considered the liberal position will affect the analysis because the direction and strength of a correlation will depend on the direction of the included issues. We have therefore, as a sensitivity test, varied which position we define as the liberal for issues that have a small argument advantage.

### Methods

We present our analysis in three steps. First, we provide a descriptive overview of the social values of newly arrived immigrants by presenting the average position on the 35 included issues for newly arrived immigrants (RQ1), as well as compare them to the average positions for the 17 included issues for native-born Swedes (RQ3).

Second, we analyse how the social values of immigrants vary with country-of-origin and individual characteristics (RQ2). We fit 35 multilevel models (one per issue) with participants nested in the country of origin. The model has the form:

$${\mathrm{Position}}_{\mathrm{ij}}=\beta_{0}+\beta_{1}X_{\mathrm{ij}}+\beta_{2}X_{j}+u_{j}+e_{\mathrm{ij}}$$

where Position_ij_ is the position on an issue of individual i from country j. X_ij_ are individual-level and X_j_ are country-level predictors. u_j_ is a random intercept for country of origin which together with the random error e_ij_ are normally distributed. All continuous variables are standardised by subtracting the mean and dividing by two standard deviations. That way the coefficients are roughly comparable to the categorical variables [72].

Based on the previous discussion on salient explanations for the social values of immigrants in both public debates and previous research, the explanatory variables in this second step are as follows.

We include three variables on the country-of-origin level to measure the three explanations of variation in social values across the globe discussed above. First, modernisation theory points towards a correlation between socioeconomic development and social values. We use the Human Development Index (HDI) as a proxy for socioeconomic development. The index is a compound of indices of health, education, and standard of living [73]. While HDI is not a perfect measure of development, it does capture the major mechanism of modernisation theory, i.e., that people adopt more liberal values in societies in that offer good health and prosperity. Second, moral foundations theory stipulates that a free exchange of opinions is necessary for the transition from conservative to liberal positions. To capture this, we measure the freedom to exchange arguments and opinions about social values in a country with the 2021 World Press Freedom Index (henceforth press freedom) constructed by Reporters Without Borders [74]. The index captures the concept of free exchange in two ways—directly because the media is in itself an important channel for public discourse, and indirectly as an approximation of how free individuals in general are to express their ideas in that society. Third, to apprehend the effect of different cultural traditions and the cultural distance between Western and Muslim countries, we measure to what degree a country is Muslim with percentage of Muslims in the population from Pew Research Center [75].

A beneficial aspect of using these three measures is that they only have a moderate correlation with each other, between 0.36–0.51 in absolute terms, meaning that they do capture different aspects of the countries of origin (full correlation table of all included independent variables in S2 Table).

On the individual level, we include gender (coded female/male), level of education (below university education/university education), grounds for residence (non-refugees/refugees including accompanying family members), religiosity (seven-point scale), and religion (non-Muslim/Muslim).

International migration is a non-random process and immigrants are not representative of their countries of origin [76, 77]. The selective nature of migration makes it important to differentiate characteristics of the country of origin and the individual and to measure their effects separately, rather than e.g., assuming that all immigrants coming from countries with high HDI are highly educated or all immigrants coming from predominantly Muslim countries are themselves Muslims. The multilevel structure of the analyses and the inclusion of variables on both the origin-country and the individual level that control for each other in our analysis achieve this.

In the third analytical step, we compare the underlying opinion structures of newly arrived immigrants and native-born Swedes (RQ3) by correlating the average positions of the two populations with each other. We present the results both for the whole analytical sample, i.e., all newly arrived immigrants, as well as for immigrants coming from different countries of origin. In the analysis of separate countries, we only include the 24 countries with at least 30 respondents in the analytical sample, to make sure that the estimates are stable and to reduce noise caused by individual outliers.

## Results

### Demographics

Before the analyses, we first present the demographic composition of the analytical sample, displayed in Table 1. Respondents come from 125 different countries of origin. Descriptive statistics based on the 125 countries of origin, immigrants in our sample come from countries scoring high on HDI, with limited press freedom, and with a 37 percent Muslim population. The full distribution of origin countries is not displayed to protect the respondents’ confidentiality; however, we do provide a distribution of region of origin.

**Table 1:** Descriptive statistics for country-of-origin and individual variables.

Variable	N	Range	Mean	SD
HDI	125	0.39–0.96	0.76	0.15
Press freedom	125	18.55–93.28	59.55	19.47
Percent Muslim	125	0–99	36.52	42.13
Europe	2174	0/1	0.41	
North America & Oceania	2174	0/1	0.04	
Africa	2174	0/1	0.09	
Asia	2174	0/1	0.42	
South America	2174	0/1	0.04	
Male	2174	0/1	0.50	
University	2174	0/1	0.45	
Refugee	2174	0/1	0.26	
Religiosity	2174	1–7	3.75	1.66
Muslim	2174	0/1	0.29	

Note: Categorical variables coded 0 = No/1 = Yes. Mean and SD weighted.

On the individual level, 50 percent of the sample is male, 45 percent have a university education of at least three years, and 26 percent are refugees or family members of refugees. Respondents answered that they are, on average, neither religious nor non-religious, and 29 percent of the sample identify as Muslim.

Since the data in Table 1 is weighted by gender, education, and region of origin, the corresponding variables have a distribution that is roughly representative of the population of newly arrived immigrants in Sweden. However, the case-wise deletion of missing answers created an analytical sample that is somewhat over-educated, and with too few respondents from Europe and too many from Asia. The proportion of refugees in the analytical sample is slightly below the proportion in the whole population. It is unknown how representative the variables of religiosity and religion are as there are no available statistics to compare to.

### Social values of newly arrived immigrants

**Fig 1:**
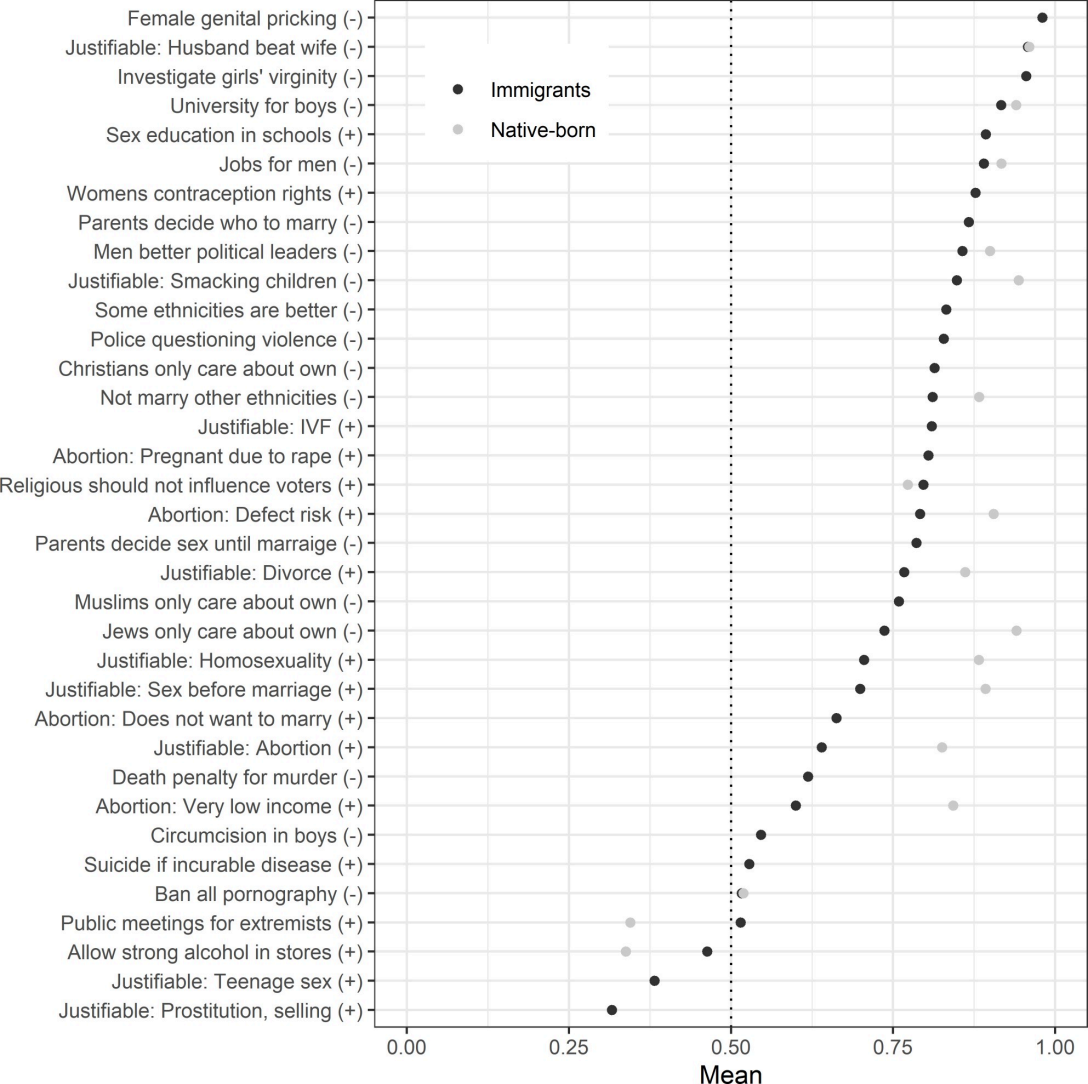
The social values of immigrants and native-born. Mean values, weighted for immigrants (and native-born when applicable). N for native-born varies across issues, see S1 Table.

Fig 1 displays the average response to each of the 35 included issues in the SIVS, standardised on a scale ranging from zero to one and weighted to be representative of the newly arrived immigrant population with regard to education, origin, and gender. The sign next to each issue indicates whether agreement (+) or disagreement (-) to the statement of the issue represents liberal values.

At the very top of Fig 1, the positions of the immigrant sample start their range from a very high liberal average position on rejecting female genital pricking; not finding it justifiable for a husband to beat his wife; and rejecting the practise of investigating girls’ virginity. At the bottom, there are the three issues on which immigrants are on average on the conservative side of the scale: being against selling strong alcohol to be sold in stores and neither finding underage teenage sex nor selling sex to be justifiable.

Comparing the values of our immigrant sample and the values of native-born Swedes collected from other sources, also visible in Fig 1, three results stand out. First, that there are some issues on which there is a near total agreement between immigrants and native-born. These issues include both when they on average take strong liberal position (not finding it justifiable for a husband to beat his wife, disagreeing that a university education is more important for boys than girls or that men should have priority to jobs), as well as the issue of banning all pornography on which both immigrants and native-born are equally split.

Second, that when the populations differ, native-born Swedes are most often more liberal. The largest differences are that native-born are more liberal on the issues of abortion due to very low income, of Jews only care about their own kind, and if it is justifiable with sex before marriage. The two issues on which immigrants are clearly more liberal than native-born Swedes are also the two issues on which the latter take an on average conservative position: against extremists being able to hold public meetings and against strong alcohol to be sold in stores (the government has a retail alcohol monopoly on strong alcohol in Sweden).

As noted, the SIVS is built on a non-probability sample, with a risk that the self-selection into participation is correlated with social values, specifically with more liberal values. To ascertain the size of this issue, we have compared the average positions across issues in the SIVS with immigrants in the same external data sources we used to compare with native-born. While we have considerably more immigrant respondents, those data sources employ probability sampling techniques, making them a good point of comparison. S2 Fig displays the results. In short, there is not much difference between our estimates and the estimates from other sources. Since we only include newly arrived immigrants, and the other sources include all regardless of duration of stay—assuming that individuals adopt the values of the country of destination over time—this means that our sample is somewhat biased toward liberal social values.

### Variation across individuals

**Fig 2:**
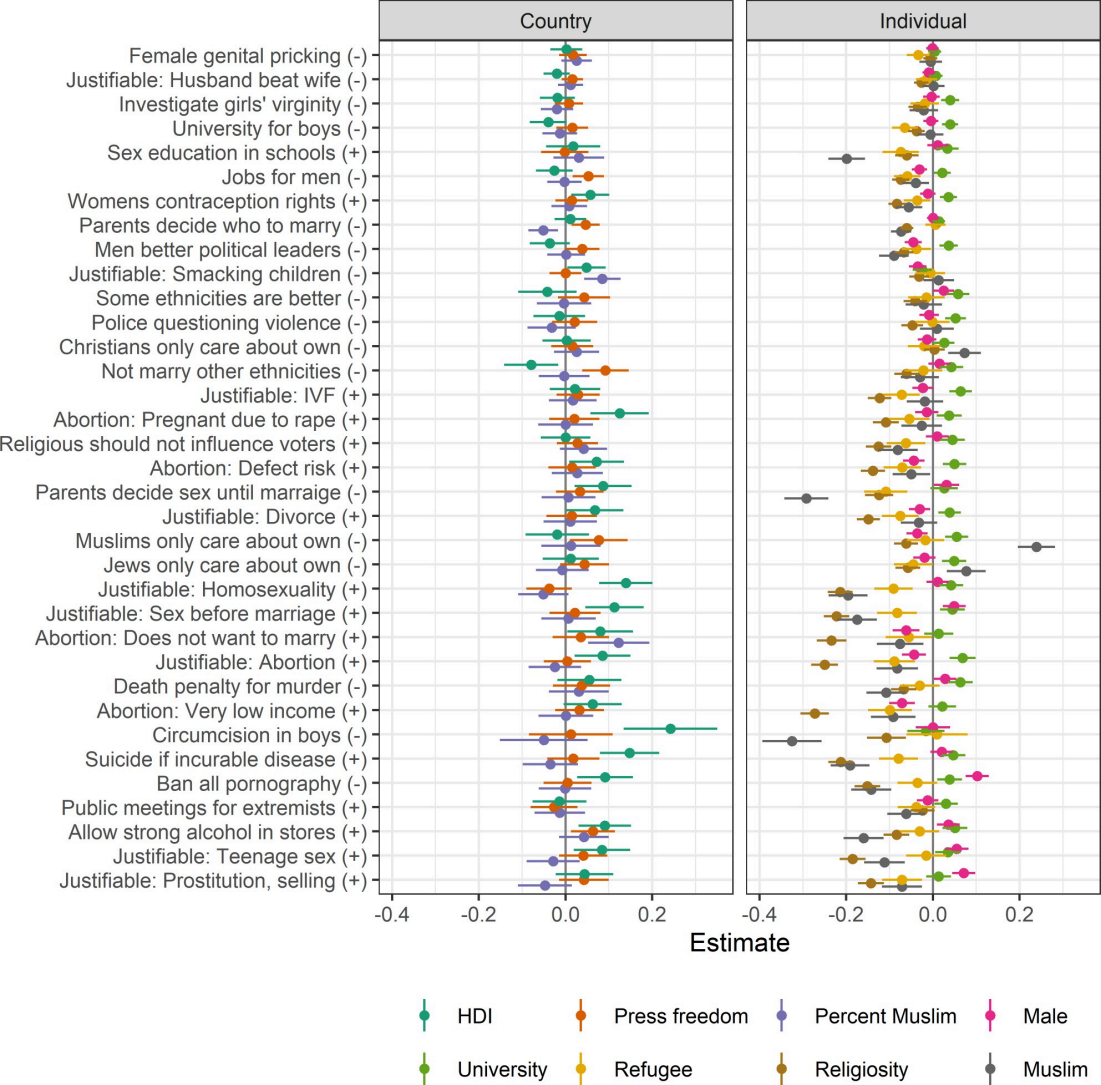
Effects of country-of-origin and individual variables on the social values of immigrants. Full results in S3 Table.

Next, we studied how the social values of immigrants correlate with country-of-origin and individual covariates. To this end, we conducted 35 multi-level regressions—one for each issue—with HDI, press freedom, and the percentage of Muslims at the country level, and gender, education, grounds for residence, religiosity, and religion on the individual level. All continuous variables are standardised so that the effect size is roughly comparable to the categorical variables. Results are shown in Fig 2.

In general, there is more variation on the individual level than on the country-of-origin level when controlling variables on the two levels for each other. Among the country-level variables, HDI has the most correlation with the included issues, with higher HDI being most often associated with more liberal social values. Issues with the largest effect size for HDI are male circumcision, suicide if incurable disease, and if homosexuality is justifiable. Level of press freedom does not have any correlation with most issues. However, when there is a discernible effect size it is always a positive correlation between more press freedom and more liberal social values, e.g., being against that family members should not marry people of other ethnicities or that Muslims only care about their own kind, and being for selling strong alcohol in stores. The percentage of Muslims in the country of origin does neither vary much with the issues nor does it have a clear direction towards either conservative or liberal social values.

Turning to the variables on the individual level. Gender doesn’t have a clear association with having liberal values. Instead, the results display more of a gender-typical pattern in that men on average take the more liberal position on issues like banning all pornography, prostitution (selling), and underage teenage sex, while at the same time taking the more conservative position on the issues abortion due the risk of a serious defect or very low income, and whether men make better political leaders than women. Having a high education is consistently associated with more liberal values, while being a refugee is quite consistent with having more conservative values. However, the effect sizes for these three variables are small.

The level of religiosity, regardless of religion, stands out as a variable with both relatively large effects and a consistent pattern in that more religious immigrants also have more conservative social values. This pattern is particularly prominent for issues related to abortion. Religious immigrants consider abortion to be much less justifiable, and that abortion due to very low income or because the woman doesn’t want to marry the man to be much more wrong.

Muslim immigrants display, on average, more conservative values than immigrants with other religious identities, though the pattern is more complex than that of religiosity. On some issues, Muslim immigrants are more conservative than other immigrants. Besides supporting male circumcision, Muslim immigrants are also considerably more conservative on issues such as parents’ say in their children’s engaging in pre-marital sex, sex education in schools, and homosexuality. On other issues, they do however not differ much from other immigrants, e.g., if divorce is justifiable, if it is justifiable for a husband to beat his wife, or if a university education is more important for a boy than a girl. Additionally, Muslim immigrants have less xenophobic values, on average disagreeing more with the statements that Christians, Jews, or Muslims only care about their own kind.

### Opinion structures

As discussed in the Introduction, we must separate between the differences in average support for issues, and differences in the underlying opinion structures between populations. As noted, it is entirely possible for two populations, e.g., immigrants and native-born, to have differences in average support for a position on an issue, but simultaneously have the same underlying opinion structure.

**Fig 3:**
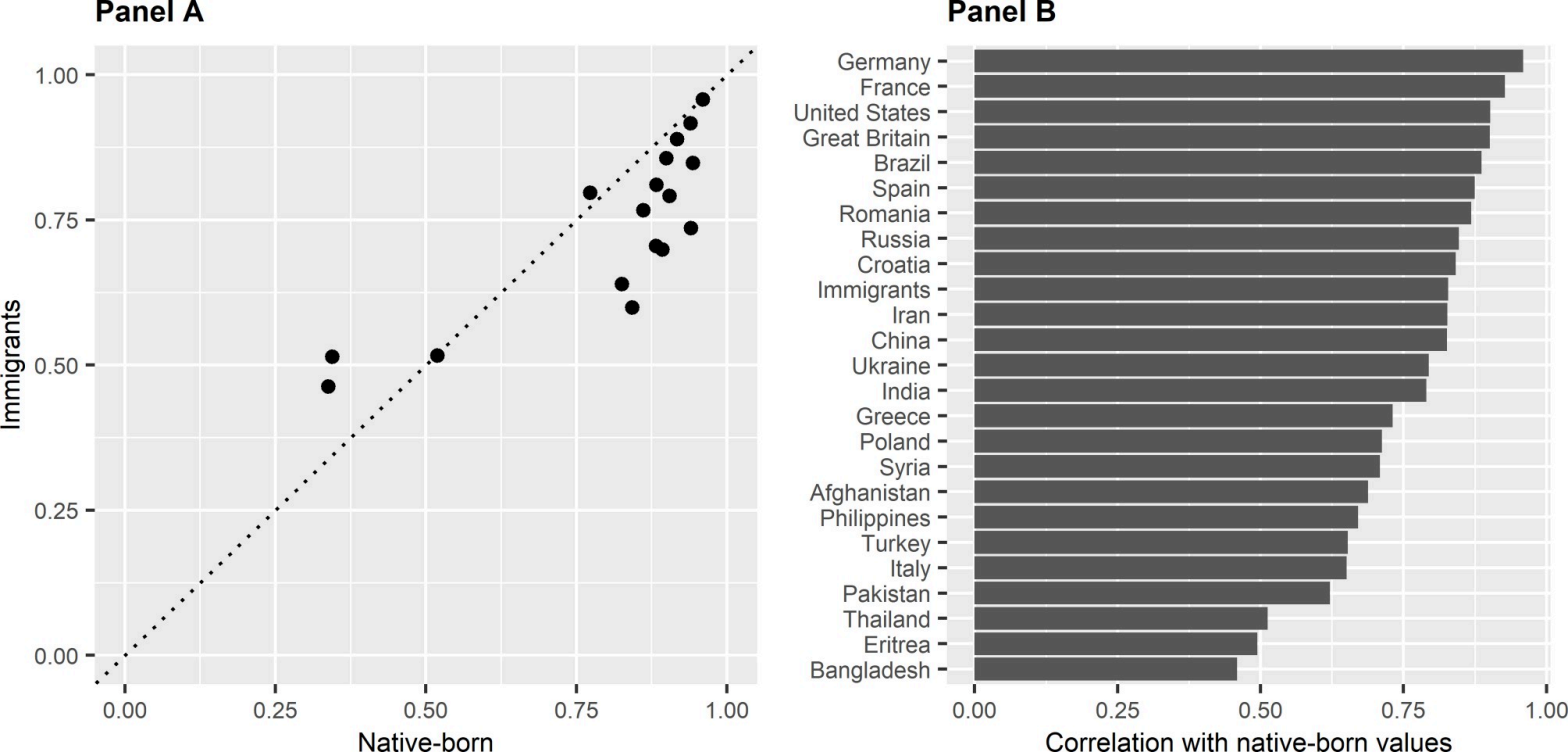
Correlation of social values between immigrants and native-born.

Panel A in Fig 3 displays the same average positions for both the immigrants and native-born Swedes on the seventeen issues for which we also have a statistic on the Swedish population as in Fig 1, this time arranged as a scatterplot. It shows that there is a common opinion structure, i.e., that the there is a large correlation between the average opinions of the two populations.

Panel B in Fig 3 displays this correlation between immigrants and native-born, as well as between immigrants in the sample from the origin countries with at least 30 respondents, respectively, and native-born. The underlying opinion structures are positively correlated between native-born and immigrants from all included countries of origin in the sample. For the entire immigrant sample, the correlation is the very large 0.83. For individual countries, the strength varies from moderate (Bangladesh 0.46) to extreme (Germany 0.96). Importantly, the correlation is never negative or even close to zero. The result is in this way clear, immigrants, regardless of origin, and native-born Swedes have the same underlying opinion structure.

The definition of which direction that is liberal for an issue affects the correlations in Fig 3, and therefore the analysis of opinion structure. Four issues have both a small argument advantage, below 0.10 in either direction, and are present in Fig 3: *Allow strong alcohol in stores (+)*, *Ban all pornography (–)*, *Abortion*: *Defect risk (+)*, and *Abortion*: *Very low income (+)*. To test the sensitivity of the direction of the issue scales for these four variables, the correlation analysis was re-run with all possible combinations of the liberal direction for these four issues, yielding a total of sixteen different models including the original. Across these models, the correlation varies between 0.79 and 0.93, i.e., that the specific direction of these four variables does not alter our conclusion that the opinion structure for immigrants and native-born is the same.

## Discussion

The purpose of this paper has been to describe the social values of newly arrived immigrants in Sweden, and to compare them with the social values of native-born Swedes. It is imperative to accurately measure and understand the social values of immigrants because of the relatively large immigration to Sweden and Europe during the last years and the concerns that immigrants have fundamentally different social values from native-born. To reach our purpose, we surveyed a large sample of newly arrived immigrants in Sweden and measured their social values by asking their opinion on 35 different moral issues covering topics such as gender equality, sexual and reproductive rights, violence, xenophobia.

We posited three research questions. First, what are the social values of newly arrived immigrants? Here, we found that immigrants had varying positions on different issues but almost always on the liberal side of an issue. On average, our immigrant sample is against violence, for gender equality, for sexual and reproductive rights, and against xenophobia. Using the included issues as an operationalisation of the concept of social values, we can conclude that the newly arrived immigrants in our sample have liberal, rather than conservative, social values.

Our second research question was how their social values correlate with country-of-origin and individual characteristics. In this analysis (Fig 2) the variables of the country-of-origin level had less explanatory power compared to variables on the individual level. In other words, immigrants’ countries of origin, come from, or the countries, offer little information about immigrants’ social values.

Among the individual traits, having a university education had a stable correlation with being more liberal and being a refugee had a stable correlation with being more conservative across the included issues, albeit with small effect sizes. Being more religious, regardless of religion, had the most consistent and largest correlation with more conservative social values. For these three variables, results are in line with previous results.

As for gender, there were no clear indication that either male or female immigrants are more liberal or conservative than the other. Instead, we saw a gendered pattern with men being more liberal on some issues, and more conservative on others. Previous research has mainly concluded that immigrant men are more conservative than women [28, 31]. By including a substantially larger set of issues compared to previous research, our study offers a more comprehensive picture of the dynamics of social values and their interplay with demographic variables.

Our results indicate, in line with previous research discussed in the introduction, that Muslims, as compared to respondents of other religions, indeed take a more conservative position on many of the included issues. Our results do however also indicate a more complex pattern than Muslim immigrants just being more conservative than other immigrants. There are some issues, such as those connected to sexual practises other than in a heterosexual marriage and especially if parents can decide if their children should wait with sex before marriage, on which Muslims take distinctly more conservative positions. But there are also other, such as issues connected to violence and divorce, on which there is no difference between Muslims and other immigrants.

These findings iterate the importance of using a breadth of issues to measure social values, as an operationalisation using too few issues may lead to erroneous conclusion. We therefore caution against using only one or a handful of issues as a point of departure for a broader narrative about Muslim versus Western values. As our results indicate, one of the most common single issues to use—homosexuality—is also one of the issues where Muslims are the most conservative compared to other immigrants. Using this issue as a proxy for social values generally will therefore systematically misrepresent this group as more conservative than they really are.

Some previous results indicate that Muslims in Western societies exhibit more antisemitic beliefs compared with individuals of other religions [78–80], and this has been one additional reason for concern of large Muslim immigration to Sweden [81]. As displayed in Fig 1, there is a considerable gap between immigrants and native-born on how much individuals agree with the statement that Jews only care about their own kind. It was in fact one of the issues with the largest gap between the two populations. There is, however, no sign of it being specifically Muslims that are responsible for this gap. On the contrary, as our result suggests, there is an association between being Muslim and disagreeing with the statement.

Our third and final research question was how immigrants’ social values compare to the social values of native-born Swedes. In the analysis of differences in average position across different issues (Fig 1), immigrants are on average somewhat more conservative than native-born Swedes. Though there is variation across the issues—on some of which the average position is almost identical for the two populations or even with immigrants being more liberal on two issues—for most of the included issues the native-born have a more liberal stance.

However, immigrants being somewhat more conservative than native-born is not the same as the two populations having fundamentally different social values. As shown in Fig 1, immigrants take on average the liberal position on 32 out of 35 issues. Of the remaining three issues where immigrants are on average on the conservative side of the spectrum, native-born Swedes are on the same side for the issue of strong alcohol in stores, and probably also for selling sex [82] though the survey question measuring native-born values is somewhat different in the latter case and it is therefore not included in Fig 1. This implies that even if Sweden would have a considerably large immigration for years to come, as long as the distribution of immigrants is roughly the same as during the last years, our results indicate any risk of Sweden drastically changing its social values or becoming a conservative country because of immigration.

We extended the comparison between immigrants and native-born by including an analysis of opinion structures using correlation analysis. Our analyses (Fig 3) showed that there is a large correlation between the average positions on issues between immigrants and native-born, i.e., the issues where native born are most liberal tend to be the ones on which immigrants are most liberal as well, and vice versa. This was true both for all newly arrived immigrants in our analytical sample, as well as for immigrants coming from a wide variety of countries. Our conclusion is that the two populations have a common opinion structure. In other words, the concerns about immigrants’ social values and the potential threat these would have to European or Western social values are greatly exaggerated, at least according to our data. This conclusion is also in line with empirical evidence that it is the country of origin, rather than the country of destination, that undergoes changes in values due to migration [83].

We have used the SIVS to study the social values of immigrants in Sweden. A core strength of the survey is the large number of included moral issues, especially on the topic of sexual and reproductive rights. Because of this, we have been able to uncover previously overlooked patterns and variations, e.g., of how gender and religion correlate with social values. It is also because of the large number of issues that a correlation analysis, measuring opinion structures, has been meaningful to make.

Though many, the included issues are not a globally exhaustive list. Compared to previous studies, the topic of governance and democracy is missing. We do not, however, expect that the inclusion of this topic would in any meaningful way alter our conclusions—as Inglehart and Norris [84] points out, the “clash of civilisations” between Western societies and the Muslim world lies not in social values concerning the governing of a society but rather on issues connected to sexual liberation, a topic we cover extensively.

More issues could also have been included within topics, though this would have additionally increased the length of an already lengthy survey. For example, the SIVS asks about the right to suicide if the person has an incurable disease but neither if suicide is generally justifiable nor about assisted euthanasia (both questions in the WVS). Also, the SIVS included only one question on antisemitism, which might explain why our result differ from previous studies, although the chosen question is moderately correlated with other measures of antisemitism [79]. Two issues where specifically taken from the Swedish political debate (and asked about by SOM), but we could always have added more moral hot topics, e.g., allowing a third legal gender or banning begging.

For future studies to include additional issues would surely reveal more nuance. However, our main results—that almost all issue positions are on the liberal side of the spectrum and that there is a large correlation between issue positions between native-born Swedes and immigrants regardless of origin country—are decisive enough to not be altered greatly by additional issues.

Similarly, our list of explanatory variables on both the origin-country and individual level, which is based on previous research, is not exhaustive. We tested three additional variables that were ultimately discarded: GDP per capita correlated with HDI to the extent that it did not add to the analysis; geographical distance and the GINI-coefficient both exhibited low explanatory power. However, we cannot rule out the existence of other important variables.

We have focused on newly arrived immigrants in Sweden, studied with a cross-sectional sample. Similarly to many previous studies, these results do not reveal how immigrants’ social values change over time in the destination country. There are good reasons to believe that that immigrants shift their values and associated practises in their new settings, even when it comes to deeply rooted traditions [85] To capture this, future research must conduct longitudinal studies. Only then can the question on immigrants socio-cultural integration into their receiving society be truly answered.

## Supporting information

S1 Table Overview of included issues. (PDF)

S2 Table Correlations between independent variables. (PDF)

S3 Table Full results for the multi-level regressions. (PDF)

S1 Fig Liberal advantage of issue positions. (TIFF)

S2 Fig Comparing immigrants’ social values: SIVS and external data sources. (TIFF)
