# Supplementary material for: The social values of newly arrived immigrants in Sweden
Source: PLoS One. 2022 Nov 22;17(11):e0278125. doi: 10.1371/journal.pone.0278125 (PMC9681117; doi:10.1371/journal.pone.0278125)
Supplement: S1 Table — (PDF) [file pone.0278125.s001.pdf]

S1 Table. Overview of included issues

**Issue**	**Question(s) in SIVS**	**External data source (N natives)**
Female genital pricking (-)	*Below are some non-medical practices that are performed on children and young people in certain cultures. How acceptable do you think the following practices are? Symbolic branding of the genital skin of girls, without removing tissue.*	
Justifiable: Husband beat wife (-)	*For each of the following alternatives, please indicate whether you think that it can never be justified, always be justified or something in between. (Justifying something means you think it can be right.) Scale 1-10, where 1 means "Never justifiable" and 10 "Always justifiable". For a man to beat his wife*	WVS (1046)
Investigate girls' virginity (-)	*Below are some non-medical practices that are performed on children and young people in certain cultures. How acceptable do you think the following practices are? To investigate and determine a girl's/woman's virginity.*	
University for boys (-)	*What do you think about the following statements using the following scale: strongly disagree - strongly agree. A university education is more important for a boy than for a girl.*	EVS/WVS (1026)
Sex education in schools (+)	*Are you for or against sex education in schools?*	
Jobs for men (-)	*What do you think about the following statements using the following scale: strongly disagree - strongly agree. When jobs are scarce, men should have more right to a job than women.*	EVS/WVS (1026)
Women's contraception rights (+)	*Do you think a woman should have the right to use contraception?*	
Parents decide who to marry (-)	Combination of *If you have a daughter, who should decide whom she should marry?* and *If you have a son, who should decide whom he should marry?*	
Men better political leaders (-)	*What do you think about the following statements using the following scale: strongly disagree - strongly agree. On the whole, men make better political leaders than women do.*	EVS/WVS (1026)
Justifiable: Smacking children (-)	*For each of the following alternatives, please indicate whether you think that it can never be justified, always be justified or something in between. (Justifying something means you think it can be right.) Scale 1-10, where 1 means "Never justifiable" and 10 "Always justifiable". Parents smacking their children*	WVS (1046)
Some ethnicities are better (-)	*You will now be asked to read a series of statements. Mark which ones you believe are true and which ones you believe are false. Some ethnicities are better than others.*	

Police questioning violence (-)	*It may be OK for the police to beat someone who is being questioned on suspicion of murder.*	
Christians only care about own (-)	*You will now be asked to read a series of statements. Mark which ones you believe are true and which ones you believe are false. Christians only care about their own kind.*	
Not marry other ethnicities (-)	*You will now be asked to read a series of statements. Mark which ones you believe are true and which ones you believe are false. I don't want anyone in my family to marry people of certain ethnicities*	ESS (1554)
Justifiable: IVF (+)	*For each of the following alternatives, please indicate whether you think that it can never be justified, always be justified or something in between. (Justifying something means you think it can be right.) Scale 1-10, where 1 means "Never justifiable" and 10 "Always justifiable". Assisted fertilization or in vitro fertilization (IVF)*	
Abortion: Pregnant due to rape (+)	*Do you think it is wrong to have an abortion in the following situations? If the woman has become pregnant due to rape*	
Religious should not influence voters (+)	*Religious leaders should not try to influence how people vote in elections.*	ISSP IV (1630)
Abortion: Defect risk (+)	*Do you think it is wrong to have an abortion in the following situations? If there is a strong chance of serious defect in the baby*	ISSP III (1024)
Parents decide sex until marriage (-)	Combination of *Should the parents decide that their daughter wait with sex until she is married?* and *Should the parents decide that their son wait with sex until he is married?*	
Justifiable: Divorce (+)	*For each of the following alternatives, please indicate whether you think that it can never be justified, always be justified or something in between. (Justifying something means you think it can be right.) Scale 1-10, where 1 means "Never justifiable" and 10 "Always justifiable". Divorce*	EVS/WVS (1026)
Muslims only care about own (-)	*You will now be asked to read a series of statements. Mark which ones you believe are true and which ones you believe are false. Muslims only care about their own kind.*	
Jews only care about own (-)	*You will now be asked to read a series of statements. Mark which ones you believe are true and which ones you believe are false. Jews only care about their own kind.*	ADL (501)
Justifiable: Homosexuality (+)	*For each of the following alternatives, please indicate whether you think that it can never be justified, always be justified or something in between. (Justifying something means you think it can be right.) Scale 1-10, where 1 means "Never justifiable" and 10 "Always justifiable". Homosexuality*	EVS/WVS (1026)
Justifiable: Sex before marriage (+)	*For each of the following alternatives, please indicate whether you think that it can never be justified, always be justified or something in between. (Justifying something means*	WVS (1046)

	you think it can be right.) Scale 1-10, where 1 means "Never justifiable" and 10 "Always justifiable". Having casual sex before marriage	
Abortion: Does not want to marry (+)	*Do you think it is wrong to have an abortion in the following situations? If the woman is not married and does not want to marry the man*	
Justifiable: Abortion (+)	*For each of the following alternatives, please indicate whether you think that it can never be justified, always be justified or something in between. (Justifying something means you think it can be right.) Scale 1-10, where 1 means "Never justifiable" and 10 "Always justifiable". Abortion*	EVS/WVS (1026)
Death penalty for murder (-)	*People convicted of murder should be sentenced to death.*	
Abortion: Very low income (+)	*Do you think it is wrong to have an abortion in the following situations? If the family has a very low income and cannot afford any more children*	ISSP IV (1630)
Circumcision in boys (-)	*Below are some non-medical practices that are performed on children and young people in certain cultures. How acceptable do you think the following practices are? Circumcision of boys where the foreskin is removed.*	
Suicide if incurable disease (+)	*You have the right to end your life if you have an incurable disease.*	
Ban all pornography (-)	*Below are two suggestions that have been found in the political debate. What is your opinion on each of them? Ban all forms of pornography*	SOM (8333)
Public meetings for extremists (+)	*There are some people whose views are considered extreme by the majority. Consider people who want to overthrow the government by revolution. Do you think such people should be allowed to hold public meetings to express their views?*	ISSP IV (1630)
Allow strong alcohol in stores (+)	*Below are two suggestions that have been found in the political debate. What is your opinion on each of them? Allow the sale of strong beer, wine and spirits in supermarkets*	SOM (8333)
Justifiable: Teenage sex (+)	*For each of the following alternatives, please indicate whether you think that it can never be justified, always be justified or something in between. (Justifying something means you think it can be right.) Scale 1-10, where 1 means "Never justifiable" and 10 "Always justifiable". Underage teenagers having casual sex*	
Justifiable: Prostitution, selling (+)	*For each of the following alternatives, please indicate whether you think that it can never be justified, always be justified or something in between. (Justifying something means you think it can be right.) Scale 1-10, where 1 means "Never justifiable" and 10 "Always justifiable". Prostitution, to sell one’s body for money*	
