# Supplementary material for: The social values of newly arrived immigrants in Sweden
Source: PLoS One. 2022 Nov 22;17(11):e0278125. doi: 10.1371/journal.pone.0278125 (PMC9681117; doi:10.1371/journal.pone.0278125)
Supplement: S2 Table — (PDF) [file pone.0278125.s002.pdf]

S2A Table. Correlations between independent variables: country level

	1	2	3
1. HDI	-		
2. Press freedom	0.51	-	
3. Percent Muslim	-0.36	-0.48	-

S2B Table. Correlations between independent variables: individual level

	1	2	3	4	5	6	7	8
1. HDI	-							
2. Press freedom	0.70	-						
3. Percent Muslim	-0.64	-0.61	-					
4. Male	0.00	0.02	0.03	-				
5. University	0.25	0.14	-0.22	0.06	-			
6. Refugee	-0.59	-0.55	0.60	0.01	-0.34	-		
7. Religiosity	-0.40	-0.25	0.25	-0.11	-0.18	0.20	-	
8. Muslim	-0.51	-0.44	0.77	-0.02	-0.21	0.49	0.30	-
