# Supplementary material for: The social values of newly arrived immigrants in Sweden
Source: PLoS One. 2022 Nov 22;17(11):e0278125. doi: 10.1371/journal.pone.0278125 (PMC9681117; doi:10.1371/journal.pone.0278125)
Supplement: S3 Table — (PDF) [file pone.0278125.s003.pdf]

S3 Table. Full results for the multi-level regressions

Issue	Effects							
	**HDI**	**Press freedom**	**Percent Muslim**	**Male**	**University**	**Refugee**	**Religiosity**	**Muslim**
Female genital pricking (-)	0.00<br>(0.00)	0.02<br>(0.02)	0.03<br>(0.03)	0.00<br>(0.00)	0.00<br>(0.00)	-0.03<br>(-0.03)	-0.01<br>(-0.01)	-0.01<br>(0.01)
Justifiable: Husband beat wife (-)	-0.02<br>(-0.02)	0.02<br>(0.02)	0.01<br>(0.01)	-0.01<br>(-0.01)	0.01<br>(0.01)	-0.01<br>(-0.01)	-0.03<br>(-0.03)	0.00<br>(0.01)
Investigate girls' virginity (-)	-0.02<br>(-0.02)	0.01<br>(0.01)	-0.02<br>(-0.02)	0.00<br>(0.00)	0.04<br>(0.04)	-0.02<br>(-0.02)	-0.04<br>(-0.04)	-0.02<br>(0.02)
University for boys (-)	-0.04<br>(-0.04)	0.02<br>(0.02)	-0.01<br>(-0.01)	0.00<br>(0.00)	0.04<br>(0.04)	-0.06<br>(-0.06)	-0.04<br>(-0.04)	-0.01<br>(0.02)
Sex education in schools (+)	0.02<br>(0.02)	0.00<br>(0.00)	0.03<br>(0.03)	0.01<br>(0.01)	0.03<br>(0.03)	-0.07<br>(-0.07)	-0.06<br>(-0.06)	-0.20<br>(0.02)
Jobs for men (-)	-0.03<br>(-0.03)	0.05<br>(0.05)	0.00<br>(0.00)	-0.03<br>(-0.03)	0.02<br>(0.02)	-0.06<br>(-0.06)	-0.07<br>(-0.07)	-0.04<br>(0.02)
Women's contraception rights (+)	0.06<br>(0.06)	0.01<br>(0.01)	0.01<br>(0.01)	-0.01<br>(-0.01)	0.04<br>(0.04)	-0.04<br>(-0.04)	-0.08<br>(-0.08)	-0.06<br>(0.02)
Parents decide who to marry (-)	0.01<br>(0.01)	0.05<br>(0.05)	-0.05<br>(-0.05)	0.00<br>(0.00)	0.01<br>(0.01)	0.01<br>(0.01)	-0.06<br>(-0.06)	-0.07<br>(0.01)
Men better political leaders (-)	-0.04<br>(-0.04)	0.04<br>(0.04)	0.00<br>(0.00)	-0.05<br>(-0.05)	0.04<br>(0.04)	-0.04<br>(-0.04)	-0.07<br>(-0.07)	-0.09<br>(0.02)
Justifiable: Smacking children (-)	0.05<br>(0.05)	0.00<br>(0.00)	0.09<br>(0.09)	-0.03<br>(-0.03)	-0.02<br>(-0.02)	-0.01<br>(-0.01)	-0.03<br>(-0.03)	0.01<br>(0.02)
Some ethnicities are better (-)	-0.04<br>(-0.04)	0.04<br>(0.04)	0.00<br>(0.00)	0.02<br>(0.02)	0.06<br>(0.06)	-0.01<br>(-0.01)	-0.04<br>(-0.04)	-0.02<br>(0.02)
Police questioning violence (-)	-0.01<br>(-0.01)	0.02<br>(0.02)	-0.03<br>(-0.03)	-0.01<br>(-0.01)	0.05<br>(0.05)	0.00<br>(0.00)	-0.05<br>(-0.05)	0.01<br>(0.02)
Christians only care about own (-)	0.00<br>(0.00)	0.02<br>(0.02)	0.03<br>(0.03)	-0.01<br>(-0.01)	0.03<br>(0.03)	-0.02<br>(-0.02)	0.00<br>(0.00)	0.07<br>(0.02)
Not marry certain ethnicities (-)	-0.08<br>(-0.08)	0.09<br>(0.09)	0.00<br>(0.00)	0.02<br>(0.02)	0.04<br>(0.04)	-0.02<br>(-0.02)	-0.06<br>(-0.06)	-0.03<br>(0.02)

Justifiable: IVF (+)	0.02	0.03	0.02	-0.02	0.06	-0.07	-0.12	-0.02
	(0.02)	(0.03)	(0.02)	(-0.02)	(0.06)	(-0.07)	(-0.12)	(0.02)
Abortion: Pregnant due to rape (+)	0.12	0.02	0.00	-0.01	0.04	-0.05	-0.11	-0.03
	(0.12)	(0.02)	(0.00)	(-0.01)	(0.04)	(-0.05)	(-0.11)	(0.02)
Religious should not influence voters (+)	0.00	0.03	0.04	0.01	0.05	-0.06	-0.13	-0.08
	(0.00)	(0.03)	(0.04)	(0.01)	(0.05)	(-0.06)	(-0.13)	(0.02)
Abortion: Defect risk (+)	0.07	0.02	0.03	-0.04	0.05	-0.07	-0.14	-0.05
	(0.07)	(0.02)	(0.03)	(-0.04)	(0.05)	(-0.07)	(-0.14)	(0.02)
Parents decide sex until marriage (-)	0.09	0.03	0.01	0.03	0.03	-0.11	-0.12	-0.29
	(0.09)	(0.03)	(0.01)	(0.03)	(0.03)	(-0.11)	(-0.12)	(0.03)
Justifiable: Divorce (+)	0.07	0.01	0.01	-0.03	0.04	-0.08	-0.15	-0.03
	(0.07)	(0.01)	(0.01)	(-0.03)	(0.04)	(-0.08)	(-0.15)	(0.02)
Muslims only care about own (-)	-0.02	0.08	0.01	-0.04	0.05	-0.02	-0.06	0.24
	(-0.02)	(0.08)	(0.01)	(-0.04)	(0.05)	(-0.02)	(-0.06)	(0.02)
Jews only care about own (-)	0.01	0.04	-0.01	-0.02	0.05	-0.05	-0.06	0.08
	(0.01)	(0.04)	(-0.01)	(-0.02)	(0.05)	(-0.05)	(-0.06)	(0.02)
Justifiable: Homosexuality (+)	0.14	-0.04	-0.05	0.01	0.04	-0.09	-0.21	-0.20
	(0.14)	(-0.04)	(-0.05)	(0.01)	(0.04)	(-0.09)	(-0.21)	(0.02)
Justifiable: Sex before marriage (+)	0.11	0.02	0.01	0.05	0.05	-0.08	-0.22	-0.17
	(0.11)	(0.02)	(0.01)	(0.05)	(0.05)	(-0.08)	(-0.22)	(0.02)
Abortion: Does not want to marry (+)	0.08	0.04	0.12	-0.06	0.01	-0.06	-0.23	-0.08
	(0.08)	(0.04)	(0.12)	(-0.06)	(0.01)	(-0.06)	(-0.23)	(0.03)
Justifiable: Abortion (+)	0.09	0.00	-0.02	-0.04	0.07	-0.09	-0.25	-0.08
	(0.09)	(0.00)	(-0.02)	(-0.04)	(0.07)	(-0.09)	(-0.25)	(0.02)
Death penalty for murder (-)	0.05	0.04	0.03	0.03	0.06	-0.03	-0.07	-0.11
	(0.05)	(0.04)	(0.03)	(0.03)	(0.06)	(-0.03)	(-0.07)	(0.02)
Abortion: Very low income (+)	0.06	0.03	0.00	-0.07	0.02	-0.10	-0.27	-0.09
	(0.06)	(0.03)	(0.00)	(-0.07)	(0.02)	(-0.10)	(-0.27)	(0.03)
Male circumcision (-)	0.24	0.01	-0.05	0.00	-0.02	0.01	-0.11	-0.33
	(0.24)	(0.01)	(-0.05)	(0.00)	(-0.02)	(0.01)	(-0.11)	(0.03)
Suicide if incurable disease (+)	0.15	0.02	-0.03	0.02	0.05	-0.08	-0.21	-0.19
	(0.15)	(0.02)	(-0.03)	(0.02)	(0.05)	(-0.08)	(-0.21)	(0.02)

Ban all pornography (-)	0.09	0.01	0.00	0.10	0.04	-0.04	-0.15	-0.14
	(0.09)	(0.01)	(0.00)	(0.10)	(0.04)	(-0.04)	(-0.15)	(0.02)
Public meetings for extremists (+)	-0.01	-0.03	-0.01	-0.01	0.03	-0.04	-0.02	-0.06
	(-0.01)	(-0.03)	(-0.01)	(-0.01)	(0.03)	(-0.04)	(-0.02)	(0.02)
Allow strong alcohol in stores (+)	0.09	0.06	0.04	0.04	0.05	-0.03	-0.08	-0.16
	(0.09)	(0.06)	(0.04)	(0.04)	(0.05)	(-0.03)	(-0.08)	(0.02)
Justifiable: Teenage sex (+)	0.08	0.04	-0.03	0.06	0.03	-0.02	-0.19	-0.11
	(0.08)	(0.04)	(-0.03)	(0.06)	(0.03)	(-0.02)	(-0.19)	(0.02)
Justifiable: Prostitution	0.04	0.04	-0.05	0.07	0.01	-0.07	-0.14	-0.07
	(0.04)	(0.04)	(-0.05)	(0.07)	(0.01)	(-0.07)	(-0.14)	(0.02)

S3 Table (continued)

Issue	Variance		N	
	**Marginal R2**	**Conditional R2**	**Countries**	**Individuals**
Female genital pricking (-)	0.02	0.12	120[a]	1542[a]
Justifiable: Husband beat wife (-)	0.01	0.03	125	2174
Investigate girls' virginity (-)	0.04	0.07	123[a]	1830[a]
University for boys (-)	0.05	0.11	125	2174
Sex education in schools (+)	0.14	0.22	125	2174
Jobs for men (-)	0.12	0.17	125	2174
Women's contraception rights (+)	0.16	0.21	125	2174
Parents decide who to marry (-)	0.26	0.34	125	2174
Men better political leaders (-)	0.11	0.16	125	2174
Justifiable: Smacking children (-)	0.04	0.08	125	2174
Some ethnicities are better (-)	0.03	0.14	125	2174
Police questioning violence (-)	0.03	0.12	125	2174
Christians only care about own (-)	0.02	0.10	125	2174
Not marry certain ethnicities (-)	0.05	0.11	125	2174
Justifiable: IVF (+)	0.12	0.17	125	2174
Abortion: Pregnant due to rape (+)	0.14	0.21	125	2174
Religious should not influence voters (+)	0.09	0.13	125	2174

Abortion: Defect risk (+)	0.16	0.22	125	2174
Parents decide sex until marriage (-)	0.31	0.34	125	2174
Justifiable: Divorce (+)	0.16	0.25	125	2174
Muslims only care about own (-)	0.11	0.23	125	2174
Jews only care about own (-)	0.04	0.11	125	2174
Justifiable: Homosexuality (+)	0.39	0.42	125	2174
Justifiable: Sex before marriage (+)	0.34	0.39	125	2174
Abortion: Does not want to marry (+)	0.16	0.21	125	2174
Justifiable: Abortion (+)	0.30	0.33	125	2174
Death penalty for murder (-)	0.10	0.21	125	2174
Abortion: Very low income (+)	0.27	0.31	125	2174
Male circumcision (-)	0.30	0.39	122[a]	1797[a]
Suicide if incurable disease (+)	0.40	0.46	125	2174
Ban all pornography (-)	0.22	0.27	125	2174
Public meetings for extremists (+)	0.02	0.09	125	2174
Allow strong alcohol in stores (+)	0.18	0.22	125	2174
Justifiable: Teenage sex (+)	0.24	0.29	125	2174
Justifiable: Prostitution	0.19	0.25	125	2174

Note: Effects with associated standard errors in parentheses. Marginal R2 represents the variance explained by the fixed effects. Conditional R2 is interpreted as a variance explained by the entire model, including both fixed and random effects.

a. The survey questions for these issues contained the extra answer category “I’m not familiar with this custom”. Respondents who chose this option are excluded from the analysis for respective issue.
