# Supplementary figures and images for: The social values of newly arrived immigrants in Sweden
Source: PLoS One. 2022 Nov 22;17(11):e0278125. doi: 10.1371/journal.pone.0278125 (PMC9681117; doi:10.1371/journal.pone.0278125)

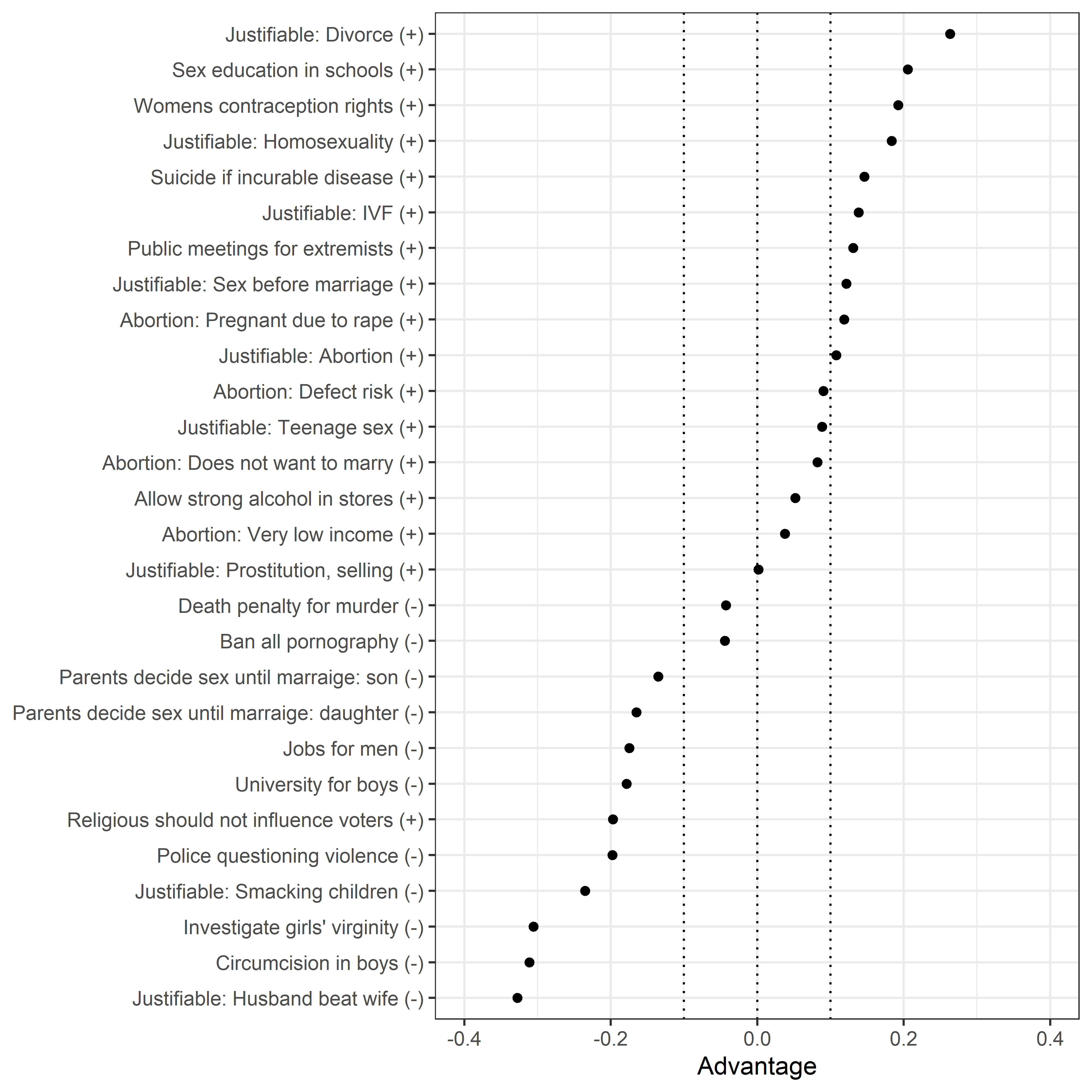

Supplement: S1 Fig — (TIFF) [file pone.0278125.s004.tiff]

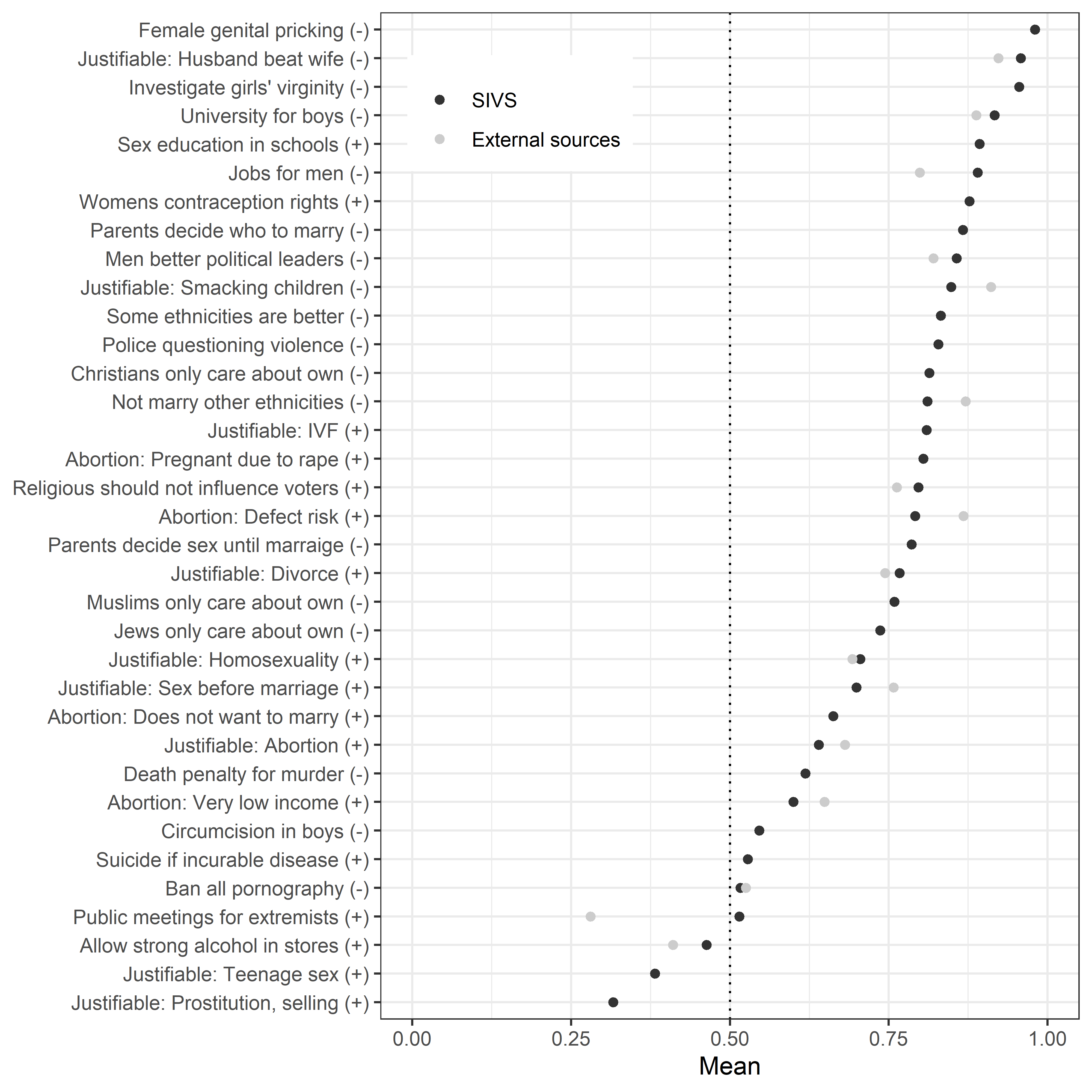

Supplement: S2 Fig — (TIFF) [file pone.0278125.s005.tiff]
